# Rituximab Therapy in Adults with Refractory Symptomatic Immune Thrombocytopenia: Long-Term Follow-Up of 15 Cases

**DOI:** 10.4274/tjh.2016.0086

**Published:** 2017-03-01

**Authors:** Fehmi Hindilerden, İpek Yönal-Hindilerden, Mustafa Nuri Yenerel, Meliha Nalçacı, Reyhan Diz-Küçükkaya

**Affiliations:** 1 Bakırköy Sadi Konuk Training and Research Hospital, Clinic of Hematology, İstanbul, Turkey; 2 İstanbul University İstanbul Faculty of Medicine, Department of Internal Medicine, Division of Hematology, İstanbul, Turkey; 3 İstanbul Bilim University Faculty of Medicine, Department of Internal Medicine, Division of Hematology, İstanbul, Turkey

**Keywords:** Immune thrombocytopenia, Rituximab, Early response, Late response, Sustained response

## Abstract

**Objective:**

This paper prospectively evaluates the long-term follow-up [mean ± standard deviation (SD) duration: 89.7±19.4 months] data of 15 patients (13 females and 2 males) with refractory symptomatic immune thrombocytopenia (ITP) treated with rituximab.

**Materials and Methods:**

Rituximab was administered at 375 mg/m2 weekly for a total of 4 doses. Complete response (CR) was defined as a platelet count of ≥100,000/mm^3^ and partial response (PR) as a platelet count of ≥30,000/mm^3^ but less than 100,000/mm^3^. Early response (ER) and late response (LR) were defined as response within 42 days and after 42 days of initiation of rituximab therapy, respectively. Sustained response (SR) was defined as response lasting for at least 6 months.

**Results:**

Mean age (±SD) at the start of rituximab was 46.6±11.3 years. Mean platelet count (±SD) prior to rituximab treatment was 17,400±8878/mm^3^. The mean time (±SD) between rituximab therapy and response to rituximab in early responders and late responders was 1.8±1.3 weeks and 10±2.8 weeks, respectively. Mean durations (±SD) of ER and LR were 51±47.2 months and 6±4.2 months, respectively. Seven of the 15 patients (46.7%) showed an initial response to rituximab (5 ER and 2 LR). The rate of SR over 6 months was 26.7% (4/15). Among the responders to rituximab, 3 (3/7, 42.9%) maintained their response 1 year after rituximab treatment and 2 (2/7, 28.6%) had ongoing response 5 years after initiation of rituximab. Two of the 7 patients (28.6%) still maintained their response 98 months after initiation of rituximab. All 5 initial responders with subsequent relapse achieved response from subsequent treatment modalities (3 CR, 2 PR).

**Conclusion:**

Our data confirm, over a long period of observation, that rituximab is safe and effective in the management of patients with chronic refractory primary ITP.

## Abstract (tr)

**Amaç:**

Çalışmamızda rituksimab ile tedavi edilen refrakter semptomatik immün trombositopeni (İTP) tanılı 15 olgunun (13 kadın ve 2 erkek) uzun süreli izlemi sonucundaki [ortalama ± SD (standart deviasyon) süresi: 89,7±19,4 ay] verileri incelenmiştir.

**Gereç ve Yöntemler:**

Rituksimab haftada bir 375 mg/m2 dozunda toplam 4 doz uygulanmıştır. Tam yanıt (TY) trombosit sayısının ≥100,000/mm^3^ olması ve parsiyel yanıt (PY) trombosit sayısının ≥30,000/mm^3^ olması fakat 100,000/mm^3^’ün altında olması olarak tanımlanmıştır. Erken yanıt (EY) ve geç yanıt (GY) ise sırasıyla rituksimab başlangıcından 42 gün içinde ve 42 gün sonra yanıt elde edilmesi olarak tanımlanmıştır. Sürekli yanıt (SY), yanıtın en az 6 ay sürmesi olarak tanımlanmıştır.

**Bulgular:**

Rituksimab tedavisinin başladığı sırada ortalama yaş (±SD) 46,6±11,3 yıldır. Rituksimab tedavisi öncesinde ortalama trombosit sayısı (±SD) 17,400±8878/mm^3^’dür. Erken ve geç yanıt edilen olgularda rituksimab başlangıcı ile yanıta kadar geçen ortalama süre (±SD) sırasıyla 1,8±1,3 hafta ve 10±2,8 hafta olarak saptanmıştır. EY ve GY elde edilen olgularda ortalama yanıt süresi sırasıyla 51±47,2 ay ve 6±4,2 aydır. On beş olgunun 7’sinde (%46,7) rituximab tedavisine başlangıçta yanıt elde edilmiştir (5 EY, 2 GY). SY oranı %26,7’dir (4/15). Rituksimab tedavisine yanıt veren olgular arasında 3’ü (3/7, %42,9) yanıtını bir yıldan fazla ve 2’si (2/7, %28,6) yanıtını 5 yıldan fazla sürdürmüştür. Yedi olgunun ikisi (%28,6) rituksimab başlangıcından 98 ay sonra halen yanıtını korumaktadır. Başlangıçta yanıt veren 5 olgunun hepsi relaps sonrasında ardışık tedavilere yanıt vermiştir (3 TY, 2 PY).

**Sonuç:**

Çalışmamız uzun bir gözlem sonucunda kronik refrakter primer İTP olgusunda rituksimab tedavisinin güvenilir ve etkili olduğunu desteklemektedir.

## INTRODUCTION

Immune thrombocytopenia (ITP) is an autoantibody-mediated disorder characterized by a plateletcount of less than 100,000/mm^3^ and increased risk of bleeding [1]. B cells play an important role in the pathophysiology of ITP, making B-cell depletion with rituximab a rational therapeutic option [2].

Glucocorticosteroids still remain the standard initial therapy for patients with symptomatic disease. Second-line treatment options include splenectomy, azathioprine, cyclosporine A, cyclophosphamide, danazol, mycophenolate mofetil, rituximab, and thrombopoietin-receptor (TPO-receptor) agonists [3]. In approximately 80% of patients, splenectomy results in response maintained at 10 years in 70% of patients, but it is associated with a life long infection risk in 1%-3% of patients [4]. Chronic refractory ITP has been defined as failure to respond to splenectomy [5]. There is no standard of care for patients with refractory or relapsing ITP after splenectomy. Although spontaneous remissions may occur in some cases, these patients carry a significant risk of bleeding and have increased morbidity and mortality [5]. Further treatment is considered in chronic refractory ITP patients with low platelet counts and bleeding symptoms [5].

Over the last decades, rituximab has been widely used to treat primary ITP patients resistant to one or more treatment lines [6,7,8,9,10,11,12,13,14,15]. Rituximab is still used off-label as a second- or third-line option in many countries. Only a few systematic reviews on the efficacy of rituximab for adult ITP patients have been published [6,16]. In the meta-analysis by Arnold et al., overall response (OR) and complete response (CR) rates with rituximab were 62.5% and 46.2%, respectively, with a median response duration of 10.5 months and a median follow-up of 9.5 months [6]. In a recent systematic review and meta-analysis including non-splenectomized ITP patients treated with rituximab, a CR rate of 46.8% was reported after a median follow-up of 6 months [16]. Khellaf et al. conducted a prospective multicenter registry of adult patients with ITP who were refractory to corticosteroids (97%), IVIG (71%), and splenectomy (10%) and were treated with rituximab [17]. After a median follow-up of 24 months, 61% showed an overall initial response and 39% had sustained response (SR) [17]. Data on the long-term efficacy of rituximab in adult ITP are limited [13,18,19,20,21]. Several studies reported SR rates ranging from 21% to 40% after a median follow-up period ranging from 2 to 5 years [13,18,19,20,21]. Here we prospectively assess the overall initial response and SR rates to rituximab in 15 chronic refractory symptomatic ITP patients with a follow-up duration of 7 years.

## MATERIALS AND METHODS

We prospectively evaluated 15 patients (13 females and 2 males) diagnosed with chronic refractory ITP, all of whom had been treated with corticosteroids and splenectomy and received various immunosuppressive agents. Rituximab was offered to the sepatients as an off-label treatment following the approval of the Ministry of Health. Informed consent for study participation was obtained from all patients. Rituximab was administered intravenously at 375 mg/m^2^ once weekly for 4 weeks between November 2007 and March 2008. Selective spleen scintigraphy was performed to rule out accessory spleens. Baseline platelet count spriortoinitial administration of rituximab and before each weekly infusion were recorded. During the follow-upperiod, platelet counts were obtained at the 1^st^, 3^rd^, 6^th^, 12^th^, 18^th^, 24^th^, 32^nd^, 40^th^, 48^th^, 56^th^, 64^th^, 72^nd^, 80^th^, 88^th^, and 96^th^ months of rituximab therapy. CR was defined as any platelet count of at least 100,000/mm^3^ and the absence of bleeding, partial response (PR) as any platelet count between 30,000 and 100,000/mm^3^ and absence of bleeding, and no response (NR) as any platelet count lower than 30,000/mm^3^ or the presence of bleeding [1]. Early response (ER) was defined as a response within 42 days of rituximab infusion and late response (LR) was defined as response occurring 42 days after initiation of rituximab. OR to rituximab was the summation of ER and LR. SR was defined as response lasting for a minimum of 6 months [11,22]. Loss of response was defined as losing response to rituximab with any platelet count lower than 30,000/mm^3^ or the presence of bleeding and need for other therapy during follow-up. Time to response was defined as time from commencement of treatment to either CR or PR. Duration of response was defined as time from CR or PR until loss of CR or PR.

### Statistical Analysis

Data were processed using SPSS 21 (University of Sussex). Characteristics of patients were described with mean ± standard deviation. Comparisons between groups were performed by chi-square test and Fisher’s exact test. The analysis of continuous variables among the groups was performed using the Mann-Whitney U test. Odds ratios are accompanied by Cornfield 95% confidence interval limits (CIs). A curve showing the proportion of patients with continuing response to rituximab was constructed by the Kaplan-Meier method. A general linear model for repeated measures was used to compare platelet values after the initiation of rituximab in responders vs. non-responders. Probability values of p<0.05 were considered significant.

## RESULTS

Patient characteristics are summarized in Table 1. There was no response to initial corticosteroid treatment in 7 patients (46.7%) and 8 patients (53.3%) lost their response to corticosteroids during follow-up. All patients were already splenectomized before rituximab therapy but 5 (33.3%) had an accessory spleen prior to rituximab infusion. The mean number of previous treatments was 3.6±1.04. The mean duration of follow-up after rituximab was 89.7±19.4 months. Seven of the patients (46.7%) showed an initial response (5 ER and 2 LR). The cumulative response rate was 46.7%. Four of the 15 patients (26.7%) achieved SR with a duration of more than 6 months. Patients with SR included 3 early responders and 1 late responder. During follow-up, 2 of the patients who obtained SR lost their response 9 months and 52 months after the initiation of rituximab, respectively. Durations of ER and LR were 51±47.2 months and 6±4.2 months, respectively. The duration of OR (ER+LR) was 38.1±44.4 months. One patient succumbed to intracranial hemorrhage and another to myocardial infarction. Patient characteristics of early and late responders are summarized in Table 2.

### Comparison of Immune Thrombocytopenia Patients According to Their Response Status to Rituximab Therapy

Clinical and laboratory features of ITP patients stratified by response status to rituximab are outlined in Table 3. The presence of comorbid diseases was more frequent in non-responders compared to responders, but the difference was not statistically significant (62.5% and 14.3%, respectively, p=0.117).

The presence of response did not correlate with actual age, age at diagnosis, age at time of splenectomy, age at initiation of rituximab, sex, hemoglobin level and platelet count at diagnosis, initial response to corticosteroids, number of previous therapies, interval between diagnosis and initiation of rituximab, and time between splenectomy and rituximab therapy (r<0.2). A weak positive correlation was detected between the presence of response and the interval between diagnosis and splenectomy (r=0.281). A moderate positive correlation was found between the presence of response and WBC count at diagnosis (r=0.464). We showed a moderate inverse correlation between the presence of response and comorbid diseases (r=-0.423).

### Clinical Course

**Figure 1:**
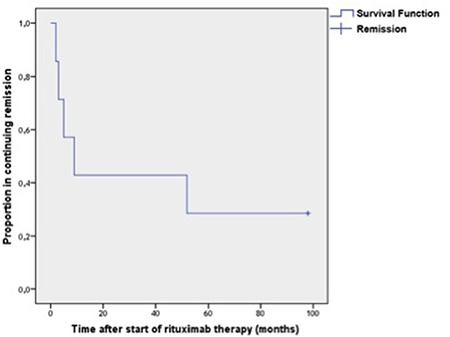
Proportion of patients with ongoing response during long-term follow-up. Two of the 7 patients (28.6%) still maintained their response 98 months after initiation of rituximab.

**Figure 2:**
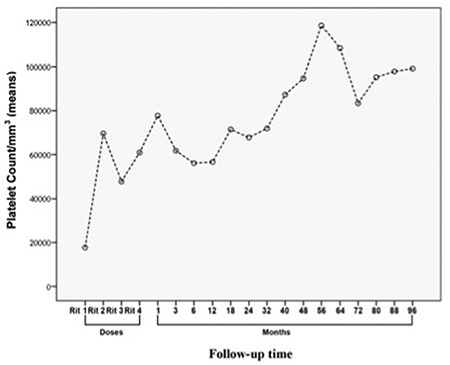
Mean platelet counts in the whole study group after initiation of rituximab. Relapsed patients and non-responders were treated with various other therapies. Mean platelet counts at times of first, second, third, and fourth doses of rituximab and 1^st^, 3^rd^, 6^th^, 12^th^, 18^th^, 24^th^, 32^no^, 40^th^, 48^th^, 56^th^, 64^th^, 72^nd^, 80^th^, 88^th^, and 96^th^ months of rituximab (±SD) were 17,400±8878/mm^3^, 70,666±122,495/mm^3^, 42,266±53,518/mm^3^, 53,533±79,974/mm^3^, 68,733±95,213/mm^3^, 54,333±81,260/mm^3^, 50,400±85,816/mm^3^, 50,266±79,408/mm^3^, 62,666±110,205/mm^3^, 59,880±116,443/mm^3^, 62,920±103,727/mm^3^, 76,746±101,374/mm3^3^, 94,707±103,763/mm^3^, 118,607±128,846/mm^3^, 108,315±119,597/mm^3^, 83,384±107,987/mm^3^, 95,230±104,396/mm^3^, 97,846±98,858/mm^3^, and 99,153±99,049/mm^3^, respectively.

**Figure 3:**
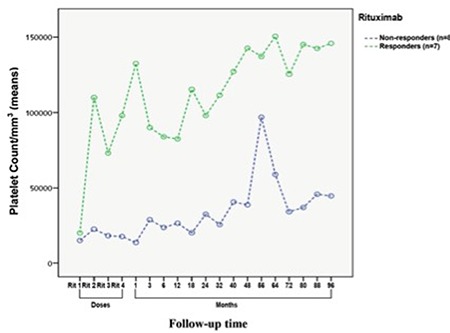
Comparison of mean platelet counts in responders and non-responders following rituximab therapy. There was a trend towards higher mean platelet counts (±SD) in initial rituximab responders (n=7) compared to non-responders (n=8) (112,201±29,008/mm^3^ vs. 33,750±31,332/mm^3^, p=0.06, odds ratio: 7.8; 95% CI 35,212-176,049). In rituximab responders mean platelet counts at times of first, second, third, and fourth doses of rituximab and 1^st^, 3^rd^, 6^th^, 12^th^, 18^th^, 24^th^, 32^nd^, 40^th^, 48^th^, 56^th^, 64^th^, 72^nd^, 80^th^, 88^th^,and 96^th^ months of rituximab (±SD) were 20,000±2905/mm^3^, 110,000±47,594/mm^3^, 73,000±18,886/mm^3^, 98,000±28,684/mm^3^, 132,714±30,811/mm^3^, 90,000±31,195/mm^3^, 83,857±33,795/mm^3^, 82,428±31,051/mm^3^, 115,428±41,586/mm^3^, 98,028±46,959/mm^3^, 111,571±39,294/mm^3^, 127,342±37,568/mm^3^, 142,657±35,001/mm^3^, 137,228±50,189/mm^3^, 150,571±43,329/mm^3^, 125,571±38,298/mm^3^, 145,142±34,753/mm^3^, 142,428±33,638/mm^3^, and 145,857±33,156/mm^3^, respectively. In non-responders mean platelet counts at times of first, second, third, and fourth doses of rituximab and 1^st^, 3^rd^, 6^th^, 12^th^, 18^th^, 24^th^, 32^nd^, 40^th^, 48^th^, 56^th^, 64^th^, 72^nd^, 80[ref:th]th[/ref], 88^th^, and 96^th^ months of rituximab (±SD) were 15,000±3138/mm^3^, 22,500±51,407/mm^3^, 18,166±20,399/mm^3^, 17,666±30,983/mm^3^, 13,666±33,279/mm^3^, 28,833±33,695/mm^3^, 23,666±36,502/mm^3^, 26,500±33,539/mm^3^, 20,166±44,918/mm^3^, 32,500±50,722/mm^3^, 25,633±42,442/mm^3^, 40,633±40,578/mm^3^, 38,766±37,805/mm^3^, 96,883±54,210/mm^3^, 59,016±46,800/mm^3^, 34,166±41,367/mm^3^, 37,000±37,537/mm^3^, 45,833±36,334/mm^3^, and 44,666±35,812/mm^3^, respectively.

Of the 7 patients with response to rituximab, 5 (3 in ER and 2 in LR) relapsed after a response duration ranging from 2 to 52 months (Figure 1). Figure 2 demonstrates the mean platelet counts in the whole population after initiation of rituximab. The mean platelet counts showed a trend to be higher in initial rituximab responders (n=7) compared to non-responders (n=8) (112,201±29,008/mm^3^ vs. 33,750±31,332/mm^3^, p=0.060, odds ratio: 7.8; 95% CI 35,212-176,049) (Figure 3).

### Status of Patients at the Final Observation

**Figure 4:**
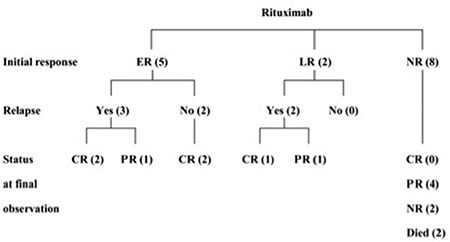
Algorithm of long-term outcome of 15 adults with immune thrombocytopenia after treatment with rituximab. ER, LR, CR, PR, and NR denote early response, late response, complete response, partial response, and no response, respectively.

Seven of the 15 patients (46.7%) showed an initial response (5 ER and 2 LR). However, 3 of the 5 early responders (20%) and all of the late responders lost their response, leaving 2 patients with long-lasting remissions with a mean follow-up of 89.7±19.4 months. Relapsed patients and patients with NR subsequently received various types of treatment, including steroids (n=12), eltrombopag (n=4), azathioprine (n=2), vincristine (n=1), danazol (n=1), IVIG (n=1), and accessory spleen operation (n=1). All of the initial 7 responders to rituximab achieved long-term remission even after relapse (5 CR, 2 PR), irrespective of subsequent treatment modalities (4: steroids, 2: eltrombopag, 1: azathioprine, 1: vincristine, 1: danazol, 1: IVIG) (Figure 4). In contrast, of the 8 non-responders, 2 patients still showed NR and 2 died (1 of intracranial hemorrhage and 1 of myocardial infarction). In total, 11 of the 15 patients (73.3%) achieved CR or PR during long-term observation with a mean follow-up time of 89.7±19.4 months.

## DISCUSSION

The algorithm for managing adult ITP has changed with the advent of rituximab and TPO-receptor agonists as options for second-line treatment. The lack of studies comparing splenectomy to other second-line therapy options presents an important dilemma. Rituximab may be a curative therapy with an initial response in 50%-60% of ITP patients and a SR of 3-5 years in 20% of patients [23]. The primary aim of our study was to evaluate the long-term efficacy of rituximab treatment in 15 patients with chronic refractory ITP. The patients described in this study had persistent, severe ITP and had received a mean of 3.6±1.04 previous therapies.

Table 4 summarizes our results as well the results of previous studies describing adult ITP patients treated with rituximab. Seven of our 15 patients achieved an initial response to rituximab (46.7%) (5 ER and 2 LR). Various studies have used different criteria to define response to ITP treatment. Our results are comparable with those of several studies that reported response rates between 40% and 55.1% [7,11,12,13,24]. However, our results are less favorable compared to several other reports [6,25,26,27,28]. In the systematic review by Arnold et al., the overall platelet count response to rituximab was 62.5% in adult ITP patients [6]. Zaja et al. in 2 different studies reported an initial response rate of 65% (13/20 patients) and 73% (27/37 patients), respectively [25,27]. The latter study hypothesized that earlier administration of rituximab enables higher rates of long-lasting response in adult ITP [27]. Moreover, our results are inferior to those of other studies reporting an OR of 66% and 82.3%, respectively [26,28].

The strength of our study lies in the long follow-up period of our patients with a mean duration of 89.7±19.4 months. We showed that 5 of the 7 responding patients (71.4%) (3/5 ER, 2/2 LR) relapsed after 2, 3, 5, 9, and 52 months, respectively. Four relapses occurred within 1 year after initial response. These findings are largely in line with previous data [9,29]. Patel et al. reported late relapses 2 years after the initiation of rituximab in adults and suggested that regular follow-up at 3-month intervals is indicated at least for the first 5 years in adults [20]. In our study, 2 adult patients showed continued response after 98 months and 1 patient relapsed 52 months after initiation of rituximab. A higher relapse rate occurred among our patients with LR compared to those with ER (100% vs. 60%). However, due to the small size of our study population, patients with ER and LR could not be compared. This issue will be the subject of our further studies.

We evaluated the relationship between clinical and laboratory variables and response to rituximab. Our results are in line with findings of several studies, which reported that splenectomy, age, sex, number of previous treatments, and pretreatment platelet count were not associated with response to rituximab [7,12]. In line with the study by Santoro et al., we demonstrated that the time between diagnosis and the start of rituximab therapy did not correlate with response to rituximab [13]. Further studies are needed to determine the best scheduling of rituximab in the course of ITP.

Considering the entire population, the SR rate in our study was 26.7% (4/15 patients) with a duration over 6 months and a mean follow-up of 89.7±19.4 months. The disease-free survival of these 4 patients at 98 months was 50%. Several other authors reported SR rates ranging from 28% to 40.5% [7,11,13,19,27]. Garcia-Chaves et al. reported a SR rate of 67% over a duration of 6 months, a finding superior to our results [30]. In that study, the mean number of therapies was 5.5 and 83% of patients had failed to respond to splenectomy [30]. In the pilot study by Arnold et al., the OR rate to rituximab at 6 months in non-splenectomized adults with newly diagnosed or relapsed primary ITP was 62.5% [31]. The aforementioned study is crucial in evaluating whether rituximab could be a valuable therapeutic alternative to splenectomy [31]. Godeau et al. evaluated the efficacy of rituximab in adult chronic ITP patients who had received at least 1 previous therapy and were potential candidates for splenectomy and reported that at 2 years the response rate was 40% [19]. Patel et al., in their study on long-term outcome after initiation of rituximab, reported that 21% of adults maintained response for at least 5 years [20]. Expert consensuses reported that 80% of ITP patients respond to splenectomy, and the response is sustained in approximately two-thirds of patients over 5-10 years [10,32]. Taking into account our results and literature review on efficacy and long-lasting response rate, we think that rituximab treatment has a role in a subset of chronic ITP patients.

## CONCLUSION

To conclude, in 15 chronic refractory ITP patients, we showed an initial response rate and SR of 46.7% and 26.7%, respectively, while 71.4% of responders subsequently relapsed in a mean follow-up period of 89.7 months. The continuing effect of rituximab had declined to 13.3% (2/15 patients) at the last follow-up. Of importance in clinical practice is the observation that all initial responders to rituximab achieved long-lasting remission even after relapse, independent of subsequent therapies. Over a very long period of immunosuppression, we did not record any serious adverse events. In the era of TPO-receptor agonists, we think that rituximab still has a role in the treatment of chronic refractory ITP. Our data, based on a long period of observation, confirm the efficacy of rituximab in refractory primary ITP. Future randomized studies including large case series are needed to determine the optimal role of rituximab and which subgroup of ITP patients can most benefit from this therapy.

## Figures and Tables

**Table 1:**
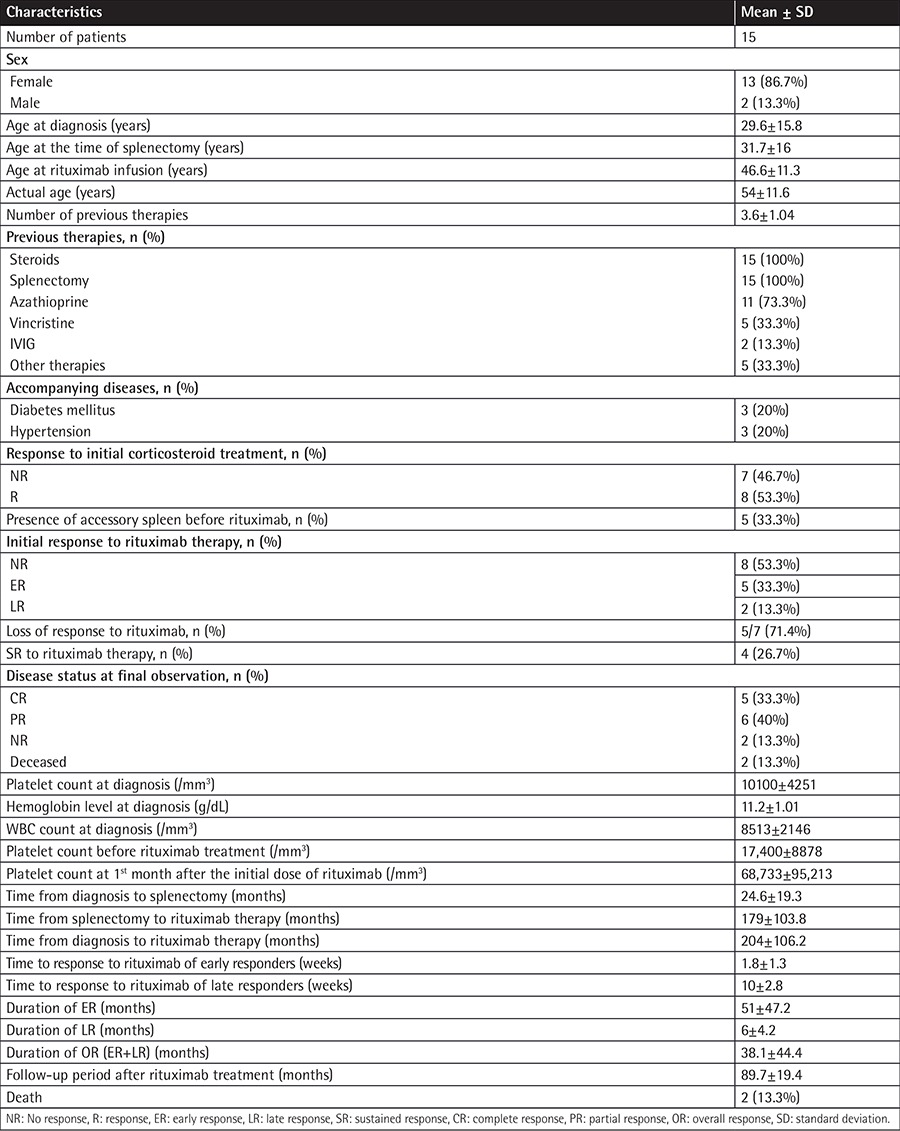
Characteristics of the patients.

**Table 2:**
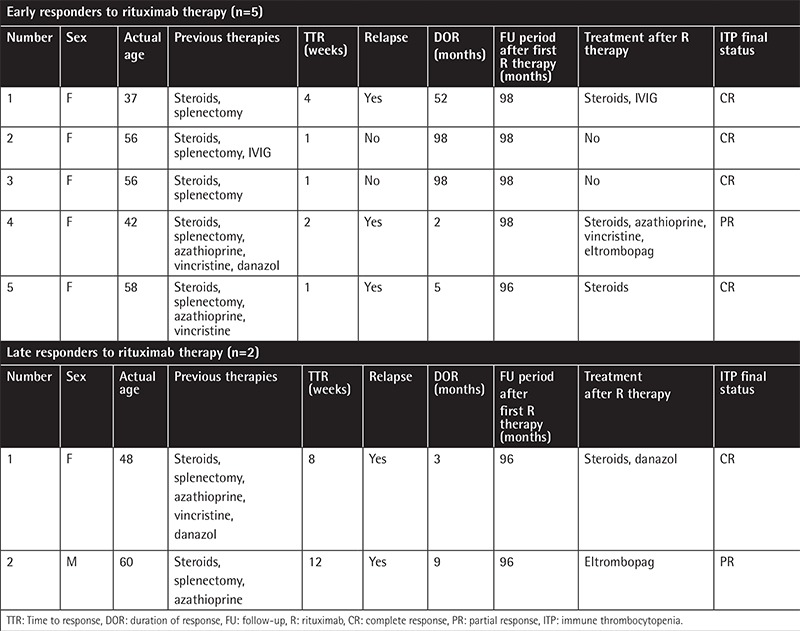
List of responders to rituximab therapy (n=7).

**Table 3:**
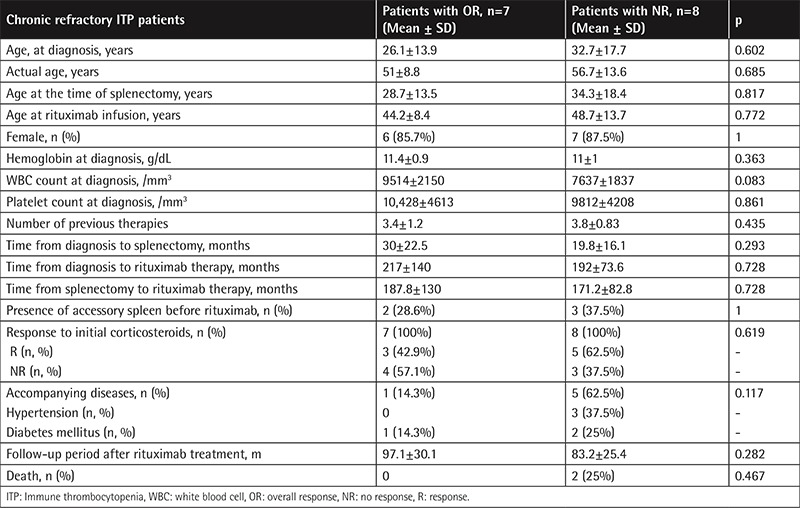
Comparison of characteristic features of immune thrombocytopenia patients stratified by response status to rituximab therapy.

**Table 4:**
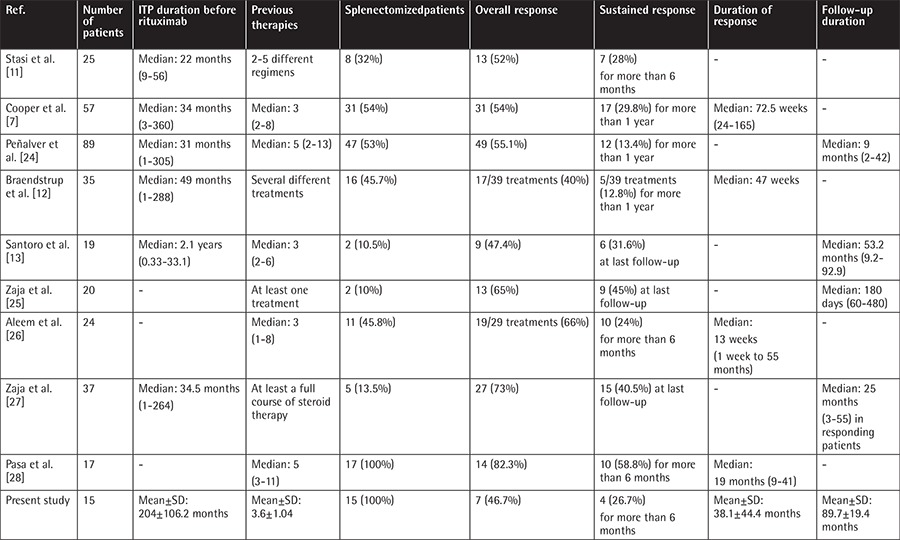
Review of the published data and our results in adults with immune thrombocytopenia treated with rituximab.
